# Associations between bullous pemphigoid and comorbidities: A Swedish nationwide cohort study of 5,738 patients

**DOI:** 10.1177/26335565261455676

**Published:** 2026-05-23

**Authors:** Zeyad Albadri, Henrike Häbel, Kristofer Thorslund, Carina Grönhagen, Oliver Seifert

**Affiliations:** 1 Division of Cell Biology, Department of Biomedical and Clinical Sciences, The Faculty of Medicine and Health Sciences, Linköping University, Linköping, Sweden; 2 Medical Statistics Unit, Department of Learning, Informatics, Management and Ethics, 27106 Karolinska Institutet, Stockholm, Sweden; 3 Dermatology and Rheumatology Clinic, 6140 Region Sörmland County, Nyköping, Sweden; 4 Department of Dermatology, 5193 Lund University, Skåne University Hospital, Malmö, Sweden; 5 Division of Dermatology and Venereology, Region Jönköping County, Jönköping, Sweden

**Keywords:** bullous pemphigoid, blistering disorders, comorbidity, epidemiology, autoimmune disorders

## Abstract

**Background:**

Bullous pemphigoid (BP) has been linked to neurological and psychiatric disorders, but no nationwide population-based study has comprehensively examined the association of various comorbidities in BP patients in Sweden.

**Objectives:**

To investigate the associations between BP and various comorbidities, comparing these conditions before and after BP diagnosis with a matched control group.

**Methods:**

A nationwide cohort study was conducted in Sweden from 2005 to 2016, including 5,738 BP cases and 17,167 age, sex and county of residence matched controls. Multivariable Cox proportional hazard regression models were used to calculate hazard ratios (HR). Univariable logistic regression assessed pre-diagnosis comorbidities, generating prevalence odds ratios (POR) with 95% confidence intervals (CI).

**Results:**

BP was associated with a significantly higher overall HR for comorbidity (HR: 2.20, 95% CI: 2.08–2.33). Before BP diagnosis, the overall comorbidity was significantly increased POR 2.72 (95% CI: 2.56–2.90). Pre-diagnosis association included dementia, Parkinson’s disease, epilepsy, amyotrophic lateral sclerosis, multiple sclerosis, schizophrenia, unipolar/bipolar disorders, suicide, diabetes, stroke, systemic lupus erythematosus, systemic sclerosis, psoriasis, lichen planus, alopecia areata, and vitiligo. After diagnosis, the overall hazard ratio (HR) for comorbidities was highest within the first year (HR 2.88, 95% CI: 2.68–3.10) and remained elevated beyond one year (HR 1.57, 95% CI: 1.44–1.71). Post-diagnosis associations remained elevated for dementia, Parkinson’s disease, epilepsy, schizophrenia, unipolar/bipolar, diabetes, psoriasis, lichen planus and autoimmune diseases such as systemic sclerosis and Sjögren’s syndrome.

**Conclusion:**

BP is strongly associated with neurodegenerative, psychiatric, autoimmune, and metabolic comorbidities before and after diagnosis, highlighting their clinical significance as predisposing and prognostic factors in BP patients.

## Introduction

Bullous pemphigoid (BP) is an autoimmune blistering skin disorder, primarily affecting the elderly, posing a significant burden due to its high morbidity and mortality rates.^1^ The condition arises from autoantibodies against BP230 (BPAG1/Dystonin) and BP180 (BPAG2/Type XVII Collagen), proteins essential for dermo-epidermal cohesion.^2^ The binding of these antibodies triggers inflammation and subepidermal blistering.^1^ Clinically, BP presents as generalized tense, itchy blisters, sometimes involving mucous membranes. However, up to 20% of cases exhibit atypical symptoms such as mild pruritus, excoriations, and eczematous or urticarial lesions without frank blistering.^3^

While the exact cause of BP remains unclear, several triggers have been linked to its onset. The incidence of BP varies widely, ranging from 2.6 to 42.8 cases per million across different populations.^4–6^ In Sweden, the incidence has increased to 7 per 100,000 individuals, representing one of the highest rates in Europe.^7^ Patients with BP have a higher mortality rate compared to healthy individuals, with 1-year mortality rates in Western populations reported at 6% to 41%.^6,8,9^ Management varies but often involves long-term systemic corticosteroids alone or combined with other immunosuppressive agents.^10^

BP has been significantly associated with a wide range of comorbidities such as hypertension, diabetes mellitus, chronic kidney disease, obstructive sleep apnoea, and psychiatric conditions such as schizophrenia and mood disorders.^11^ Neurological disorders are particularly prevalent, with BP patients facing over a tenfold increased risk compared to the general population.^11^ An American study revealed that a significant association exists between BP and neurological conditions, in which 36% of BP patients had a history of neurological diseases, with dementia and Parkinson’s disease being the most common.^12^ Furthermore, BP patients exhibit higher prevalence rates of metabolic disorders. Specifically, 53% had hypertension, 40% had diabetes mellitus, and 73% had hyperlipidaemia.^11^ The association between BP and malignancies has been inconsistent across studies. Some research indicates an increased risk of certain cancers in patients with BP, while other studies do not support this finding.^13,14^

Effective management of BP requires addressing both the disease itself and its associated comorbidities to improve patient survival and quality of life; however, it remains unclear whether these comorbidities differ from those observed in other countries. Furthermore, there is limited information on the comorbidities associated with BP patients in Sweden. Therefore, this study investigates these associations on a national scale, leveraging comprehensive data from the Swedish National Patient Register (NPR) to quantify the burden of comorbidities in patients with BP and identify potential areas for targeted intervention.

## Materials and methods

### Study design

This study is a nationwide retrospective cohort analysis conducted in Sweden. It focuses on individuals aged 18 and older who have been diagnosed with BP, identified using the International Classification of Diseases (ICD) codes, specifically ICD-10: L12.0, L12.0A, L12.0B, L12.0W, L12.8, and L12.9. Data was obtained from the NPR, which contains comprehensive medical records for all residents of Sweden, covering the period from January 1, 2005, to December 31, 2016.

Patients were followed from the date of diagnosis until the earliest of three events: emigration, death, or the study’s end on December 31, 2016. Each resident’s unique personal identification number (PIN) allowed for seamless data linkage across multiple national databases. This electronic data linkage ensured comprehensive follow-up for both the BP cohort and the matched control cohort.

### Matched control cohort

Statistics Sweden was used to collect data from the entire Swedish population, and a control cohort of individuals not diagnosed with BP was selected, up to three controls per case, matched by age, sex and county of residence. The control subjects had to be alive with no prior diagnosis of BP and contributing data at the time of the index date. We assigned a date of “pseudo diagnosis” to controls, which was the date of diagnosis of BP of the case they were matched to. This matching strategy aimed to minimize potential confounding effects from these demographic variables.

### The Swedish National Patient Register

The NPR was established by the National Board of Health and Welfare in 1964 and has provided nearly complete coverage of all inpatient care in Sweden, both public and private, since 1987. The NPR includes detailed information about patients, such as their PIN, sex, age, county of residence, and hospital identification. It also contains medical data, including one primary and up to seven secondary diagnoses, coded using the ICD-10th Edition.

Reporting for inpatient care is nearly 100% complete, while specialized outpatient care reporting covering both public and private sectors stands at around 87%.^15^ The PIN assigned to all Swedish residents allows for precise linkage of information across various registries, including the NPR. This enables the tracing of patient information both retrospectively, prior to a BP diagnosis, and prospectively, after the diagnosis. The NPR is known for its high-quality data, with a 92% positive predictive value for BP diagnoses.^16^

### Identification of comorbidities

During the study period, ICD-9 and ICD-10 codes were used to classify comorbid diseases. We included 21 diagnosis codes in the analysis, each identified by its respective ICD-9 and ICD-10 codes listed in Table 1. These comorbidities were selected not only due to their potential shared pathophysiological mechanisms with BP, including autoimmune, inflammatory, metabolic, and systemic processes, but also based on their prevalence in older populations and their prevalence in previous literature examining disease clustering in BP.

**Table 1.** ICD-9 and 10 codes for comorbidities included in the study.

Comorbidities	ICD-9	ICD-10
Diabetes Mellitus	250	E10-E14
Dementia	294	F03
Schizophrenia	295	F20
Unipolar/Bipolar Disease	296	F31
Parkinson’s Disease	332	G20
Amyotrophic lateral sclerosis	335	G12
Multiple Sclerosis	340	G35
Epilepsy	345	G40
Myasthenia Gravis	358	G70
Stroke	434	I60-I69
Psoriasis	696	L40
Lichen Planus	697	L43
Alopecia Areata	704	L63
Vitiligo	709	L80
Systemic Lupus Erythematosus	710.0	M32
Systemic Sclerosis	710.1	M34
Sjögrens Syndrome	710.2	M35
Dermatomyositis	710.3	M33
Polymyositis	710.4	M33.2
Rheumatoid Arthritis	714	M06
Suicide	E950-E959	X60-X84

ICD: International Classification of Diseases.

In this study, BP was treated as the index disease. Accordingly, the term comorbidity is used to describe medical conditions occurring in relation to BP. When referring to disease burden independent of an index diagnosis, particularly in the control population or when discussing disease clustering in older individuals, the term multimorbidity is used. This distinction was applied consistently throughout the analyses and interpretation.

### Statistics

The demographic data of the study population were analysed first. Continuous data are presented as the mean value and standard deviation (SD), and categorical variables are presented as percentages. Statistical analyses were conducted using three predefined analytical frameworks, pre-diagnosis, post-diagnosis, and overall post-diagnosis associations.

Pre-diagnosis analysis assessed the prevalence of selected conditions occurring before the BP index date. Univariable logistic regression models were used to estimate prevalence odds ratios (PORs) with 95% confidence intervals (CIs), comparing BP cases with matched controls. This analysis aimed to identify conditions potentially preceding or predisposing to BP.

Post-diagnosis analysis evaluated incident comorbidities after BP diagnosis using multivariable Cox proportional hazards regression models. Individuals with a diagnosis of the specific condition prior to the BP index date were excluded from the corresponding analysis. Hazard ratios (HRs) with 95% CIs were calculated, and analyses were stratified into events occurring within the first year after BP diagnosis and events occurring beyond one year.

Overall post-diagnosis association refers to the cumulative association between BP and incident comorbidities during the entire follow-up period after BP diagnosis, excluding individuals with a prior diagnosis of the condition of interest. Person-years at risk were calculated from the BP index date until first diagnosis of the outcome, death, emigration, or end of follow-up.

All models were adjusted for age, sex and county of residence. Standard errors in logistic regression were calculated using cluster-robust sandwich estimators. Statistical analyses were performed using STATA® version 15.1, and a two-sided significance level of 5% was applied.

## Results

### Study cohort characteristics

The study included 5,738 individuals diagnosed with BP in the NPR between 2005 and 2016, including 3,026 women (52.7%). The mean age of BP patients was 78.6 years (±12.2 SD). The total observation period for BP patients amounted to 18,022 person-years, with an average follow-up of 3.1 years (±2.9 SD). Most BP cases (83.2%, n = 4,772) were diagnosed in outpatient dermatology clinics. The control cohort comprised 17,167 randomly selected individuals from the Swedish general population. The mean age of this group was 78.7 years (±12.2 SD). Their mean follow-up duration was 4.2 years (±3.1 SD), covering a total observation period of 71,682 person-years (Table 2). During follow-up, 38% of the controls (n=6,598 out of 17,167) and 56% of the patients with BP (n=3,192 out of 5,738) died. This resulted in an approximately one-year longer mean follow-up time for the control cohort compared to the BP patients.

**Table 2.** Demographic data for patients diagnosed with bullous pemphigoid (BP) and control cohort from the general population.

​	BP	Control cohort
Subjects, *n*	5,738	17,167
Female, *n* (%)	3,026 (52.7)	9,064 (52.8)
Follow-up time, mean±SD	3.1±2.9	4.2±3.1
Age, years, mean±SD	78.6±12.2	78.7±12.2
Patients-years of observation	18,022	71,682

SD: standard deviation.

### Overall comorbidity association after a BP diagnosis

Overall, 2,712 incidents of comorbidities occurred in the BP cohort, while 5,247 incidents were observed in the controls within the 1-11 year follow-up period. Patients with BP showed a significantly increased association with several comorbidities. Overall, BP patients exhibited a higher burden of comorbid conditions compared to controls, with an HR of 2.20 (95% CI: 2.08–2.33). Regarding specific types of comorbidities, neurological and psychiatric disorders, including dementia, were notably more prevalent in BP patients. Dementia revealed a higher association with an HR of 1.59 (95% CI: 1.43–1.77). Epilepsy showed a significantly higher association, with an HR of 2.21 (95% CI: 1.67–2.92). Unipolar/Bipolar diseases were also more common, with an HR of 2.47 (95% CI: 1.35–4.49). Suicide was significantly higher in BP patients (HR: 1.35, 95% CI: 1.04–1.76).

Dermatological and autoimmune diseases showed strong associations with BP. Psoriasis had an HR of 2.16 (95% CI: 1.45–3.20), systemic sclerosis was elevated at 2.10 (95% CI: 1.40–3.14), and lichen planus exhibited the highest association, with an HR of 5.55 (95% CI: 3.14–9.79). Diabetes was also more common in BP patients (HR of 1.66, 95% CI: 1.43–1.93). A few conditions, such as systemic lupus erythematosus, polymyositis, and dermatomyositis, had very low numbers of observed cases, making it difficult to draw firm conclusions (Table 3).

**Table 3.** Overall post-diagnosis hazard ratios (HR) between bullous pemphigoid (BP) and incident comorbidities during follow-up.

Comorbidities	Observed numbers of comorbidities		HR overall (95% CI)
	BP cohort	Control cohort	
All comorbidities	2,712	5,247	**2.20 (2.08-2.33)**
Diabetes Mellitus	240	633	**1.66 (1.43-1.93)**
Dementia	465	1417	**1.59 (1.43-1.77)**
Schizophrenia	7	11	2.19 (0.85-5.68)
Unipolar/Bipolar Disease	18	27	**2.47 (1.35-4.49)**
Parkinson’s Disease	38	127	1.23 (0.85-1.78)
Amyotrophic lateral sclerosis	9	28	1.49 (0.69-3.20)
Multiple Sclerosis	3	4	2.60 (0.57-11.78)
Epilepsy	77	144	**2.21 (1.67-2.92)**
Myasthenia Gravis	2	4	2.08 (0.37-11.67)
Stroke	48	199	1.07 (0.78-1.48)
Psoriasis	39	72	**2.16 (1.45-3.20)**
Lichen Planus	30	20	**5.55 (3.14-9.79)**
Alopecia Areata	5	7	2.87 (0.90-9.18)
Vitiligo	2	3	2.89 (0.46-18.03)
Systemic Lupus Erythematosus	1	0	​
Systemic Sclerosis	37	72	**2.10 (1.40-3.14)**
Sjögrens Syndrome	9	19	1.96 (0.87-4.40)
Dermatomyositis	1	4	​
Polymyositis	0	0	​
Rheumatoid Arthritis	1	0	​
Suicide	74	224	**1.35 (1.04-1.76)**

Bold: 95% confidence interval (95% CI) does not include 1.00. ICD-10: International Classification of Diseases, tenth revision.

### Comorbidities before BP diagnosis

The total number of comorbidities observed among BP patients before they were diagnosed with BP is higher (n=3392) than among controls (n=4790). Overall, BP patients exhibit a significantly higher prevalence of comorbid conditions, with the POR at 2.72 (95% CI: 2.56-2.90) for all comorbidities combined. For specific comorbidities, the POR varies. Some of the most notable associations include neurological disorders such as Parkinson’s disease (POR: 3.73, 95% CI: 3.05-4.56), epilepsy (POR: 2.55, 95% CI: 2.10-3.10), dementia (POR: 2.75, 95% CI: 2.51-3.02), amyotrophic lateral sclerosis (ALS) (POR: 2.25, 95% CI: 1.46-3.47), and multiple sclerosis (MS), which presents the highest overall POR at 7.56 (95% CI: 4.95-11.56). Metabolic and cerebrovascular comorbidities revealed increased prevalence for diabetes (POR: 1.89, 95% CI: 1.74-2.06) and stroke (POR: 2.51, 95% CI: 1.99-3.16).

Additionally, psychiatric conditions such as schizophrenia (POR: 1.63, 95% CI: 1.06-2.50) and unipolar/bipolar disorder (POR: 1.61, 95% CI: 1.17-2.22) also show increased prevalence. Suicide risk is notably higher in BP patients, with a POR of 1.82 (95% CI: 1.52-2.18).

Interestingly, dermatological conditions such as psoriasis (POR: 2.54, 95% CI: 2.11-3.07) and lichen planus (POR: 3.51, 95% CI: 2.53-4.86) are strongly associated with BP, particularly in the period leading up to diagnosis, where the POR for lichen planus reaches 20.46 (95% CI: 8.00-52.33). Moreover, vitiligo (POR: 4.50, 95% CI: 2.17-9.34) was positively associated with BP before diagnosis, especially one year before the onset of BP (POR 26.96, 95% CI: 3.42-212.87). Additionally, alopecia areata showed a strong association before BP diagnosis (POR: 2.53, 95% CI: 1.13–5.66).

Autoimmune diseases, such as systemic lupus erythematosus (POR: 3.59, 95% CI: 1.10-11.78) and systemic sclerosis (POR: 1.34, 95% CI: 1.09-1.65), also exhibit an increased prevalence.

The likelihood of having a comorbidity within one year prior to BP diagnosis was higher for several conditions, including psoriasis (POR 7.93, 95% CI: 4.67-13.46), lichen planus (POR 20.46, 95% CI: 8.00-52.33), vitiligo (POR 26.96, 95% CI: 3.42-212.87), and amyotrophic lateral sclerosis (POR 5.99 (95% CI: 1.50-23.95)**.**

In other cases, the sample size was insufficient to conduct a robust analysis, as seen in dermatomyositis, polymyositis, and rheumatoid arthritis (Table 4).

**Table 4.** Pre-diagnosis prevalence odds ratios (PORs) for comorbidities occurring ≤1 year and >1 year before bullous pemphigoid (BP) diagnosis.

Comorbidities	Observed no. of comorbidities		POR overall (95% CI)	POR ≤ 1 year before BP (95% CI)	POR > 1 year before BP (95% CI)
	BP	Control cohort			
All comorbidities	3392	4790	**2.72 (2.56-2.90)**	**2.83 (2.50-3.20)**	**2.45 (2.29-2.61)**
Diabetes Mellitus	1035	1789	**1.89 (1.74-2.06)**	**2.15 (1.69-2.73)**	**1.82 (1.67-1.99)**
Dementia	936	1136	**2.75 (2.51-3.02)**	**2.62 (2.16-3.18)**	**2.65 (2.40-2.94)**
Schizophrenia	32	59	**1.63 (1.06-2.50)**	1.50 (0.27-8.17)	**1.64 (1.05-2.55)**
Unipolar/Bipolar Disease	58	108	**1.61 (1.17-2.22)**	1.20 (0.38-3.82)	**1.65 (1.19-2.31)**
Parkinson’s Disease	217	179	**3.73 (3.05-4.56)**	**2.77 (1.59-4.83)**	**3.87 (3.12-4.80)**
Amyotrophic lateral sclerosis	36	48	**2.25 (1.46-3.47)**	**5.99 (1.50-23.95)**	**2.00 (1.26-3.18)**
Multiple Sclerosis	75	30	**7.56 (4.95-11.56)**	1.0 (1.0-1.0)	**7.16 (4.67-10.98)**
Epilepsy	189	226	**2.55 (2.10-3.10)**	**3.60 (2.12-6.13)**	**2.41 (1.95-2.97)**
Myasthenia Gravis	5	14	1.07 (0.38-2.97)	1.00 (1.00-1.00)	1.25 (0.44-3.54)
Stroke	134	162	**2.51 (1.99-3.16)**	**2.03 (1.13-3.64)**	**2.60 (2.02-3.34)**
Psoriasis	203	244	**2.54 (2.11-3.07)**	**7.93 (4.67-13.46)**	**2.06 (1.68-2.54)**
Lichen Planus	79	68	**3.51 (2.53-4.86)**	**20.46 (8.00-52.33)**	**2.15 (1.46-3.15)**
Alopecia Areata	11	13	**2.53 (1.13-5.66)**	1.50 (0.14-16.50)	**2.72 (1.16-6.41)**
Vitiligo	18	12	**4.50 (2.17-9.34)**	**26.96 (3.42-212.87)**	**2.45 (1.01-5.91)**
Systemic Lupus Erythematosus	6	5	**3.59 (1.10-11.78)**	2.99 (0.19-47.84)	3.74 (1.00-13.94)
Systemic Sclerosis	134	300	**1.34 (1.09-1.65)**	1.62 (0.83-3.19)	**1.32 (1.06-1.64)**
Sjögrens Syndrome	27	72	​	1.00 (0.20-4.94)	1.13 (0.71-1.80)
Dermatomyositis	2	0	​	​	​
Polymyositis	1	0	​	​	​
Rheumatoid Arthritis	1	3	​	​	​
Suicide	193	322	**1.82 (1.52-2.18)**	**2.05 (1.27-3.31)**	**1.78 (1.46-2.16)**

Bold: 95% confidence interval (95% CI) does not include 1.00.

### Comorbidities after BP diagnosis

In this study, we examined comorbidities that occur after the diagnosis of BP. The findings indicate a significantly elevated association of various comorbidities in patients diagnosed with BP compared to the matched control cohort. The overall HR for developing any comorbidity within the first year of BP diagnosis is 2.88 (95% CI: 2.68-3.10), which declines but remains elevated beyond one year at 1.57 (95% CI: 1.44-1.71). Specific conditions show varying degrees of association, with some conditions exhibiting a particularly high association with BP (Table 5).

**Table 5.** Post-diagnosis hazard ratios (HR) for comorbidities occurring ≤1 year and >1 year after bullous pemphigoid (BP) diagnosis.

Comorbidities	Observed no. of comorbidities ≤ 1 year		HR overall (95% CI)	Observed no. of comorbidities > 1 year		HR overall (95% CI)
	BP	Control		BP	Control	
All comorbidities	1649	2070	**2.88 (2.68-3.10)**	1063	3177	**1.57 (1.44-1.71)**
Diabetes Mellitus	103	150	**2.47 (1.92-3.18)**	137	483	**1.35 (1.11-1.63)**
Dementia	153	300	**1.94 (1.59-2.36)**	312	1117	**1.46 (1.29-1.66)**
Schizophrenia	3	4	2.33 (0.52-10.40)	4	7	2.11 (0.61-7.25)
Unipolar/Bipolar Disease	8	7	**3.75 (1.36-10.36)**	10	20	1.96 (0.91-4.21)
Parkinson’s Disease	15	24	**2.09 (1.09-3.98)**	23	103	0.98 (0.62-1.55)
Amyotrophic lateral sclerosis	3	4	2.43 (0.54-10.86)	6	24	1.26 (0.51-3.14)
Multiple Sclerosis	1	2	1.60 (0.14-17.63)	2	2	3.71(0.51-27.19)
Epilepsy	33	32	**3.44 (2.11-5.60)**	44	112	**1.77 (1.24-2.52)**
Myasthenia Gravis	0	2	0.00 (0.00-0.00)	2	2	4.90(0.66-36.40)
Stroke	11	39	0.96 (0.49-1.88)	37	160	1.11 (0.77-1.60)
Psoriasis	15	17	**3.01 (1.50-6.03)**	24	55	**1.84 (1.13-3.00)**
Lichen Planus	19	5	**12.51(4.67-33.52)**	11	15	**3.00 (1.37-6.55)**
Alopecia Areata	2	2	3.21 (0.45-22.78)	3	5	2.71(0.64-11.53)
Vitiligo	2	1	6.86 (0.62-75.79)	0	2	​
Systemic Lupus Erythematosus	1	0	​	0	0	​
Systemic Sclerosis	11	19	2.06 (0.98-4.33)	26	53	**2.12 (1.31-3.42)**
Sjögrens Syndrome	1	5	0.61 (0.07-5.18)	8	14	**2.62 (1.08-6.39)**
Dermatomyositis	0	0	​	​	​	​
Polymyositis	0	0	​	​	​	​
Rheumatoid Arthritis	0	0	​	​	​	​
Suicide	22	41	**1.77 (1.06-2.98)**	52	183	1.23 (0.90-1.69)

Bold: 95% confidence interval (95% CI) does not include 1.00.

Among metabolic disorders, diabetes presents an increased association, with an HR of 2.47 (95% CI: 1.92-3.18) within the first year and a lower but still significant HR of 1.35 (95% CI: 1.11-1.63) beyond one year. Stroke, in contrast, does not show a significant increase. Neurological disorders also show notable associations, with dementia having an HR of 1.94 (95% CI: 1.59-2.36) within the first year and 1.46 (95% CI: 1.29-1.66) after one year. Parkinson’s disease shows an increased association initially (HR: 2.09, 95% CI: 1.09-3.98), but this association diminishes beyond the first year. Epilepsy has an HR of 3.44 (95% CI: 2.11-5.60) within the first year and remains elevated thereafter at 1.77 (95% CI: 1.24-2.52).

Psychiatric conditions display mixed association levels. Unipolar/Bipolar diseases have elevated HRs, with HRs of 3.75 (95% CI: 1.36-10.36), although wide confidence intervals suggest a degree of uncertainty. Suicide risk is significantly elevated within the first year (HR: 1.77, 95% CI: 1.06-2.98), though this association weakens over time and becomes insignificant (HR: 1.23, 95% CI: 0.90-1.69).

Dermatological diseases such as psoriasis (HR: 3.01, 95% CI: 1.50-6.03) and lichen planus (HR: 12.51, 95% CI: 4.67-33.52) show strong associations with BP in the first year, with a reduced but still significantly elevated beyond one year (HR: 1.84, 95% CI: 1.13-3.00) and (HR: 3.00, 95% CI: 1.37-6.55) respectively.

Some conditions, such as systemic sclerosis and Sjögren’s Syndrome, maintain an elevated association beyond the first year, with HRs of 2.12 (95% CI: 1.31-3.42) and 2.62 (95% CI: 1.08-6.39), respectively. Several conditions, including dermatomyositis, polymyositis, and rheumatoid arthritis, lack sufficient data to determine any associations.

## Discussion

This nationwide population-based cohort study demonstrates that BP is associated with a broad spectrum of systemic diseases, highlighting that BP extends beyond a skin-limited condition. Patients with BP exhibited a markedly higher burden of both pre-existing and incident comorbidities compared with age, sex and county of residence matched controls. These findings support the concept that BP occurs within a broader context of multimorbidity, particularly in older individuals, and emphasize the importance of viewing BP as part of a complex systemic disease profile.

Our findings confirm a substantially increased overall burden of systemic disease in patients with BP, consistent with previous large-scale epidemiological studies.^17^ The elevated prevalence of conditions prior to BP diagnosis and the increased incidence after diagnosis together indicate that BP occurs within a broader context of immune dysregulation and age-related multimorbidity rather than as an isolated dermatologic entity.

The temporal distinction between conditions occurring before and after BP diagnosis provides important clinical insight. Comorbidities identified prior to BP may represent shared pathophysiological mechanisms, immune priming, or systemic vulnerability that predisposes to BP development. In contrast, conditions arising after diagnosis may reflect the combined effects of chronic inflammation, treatment exposure, functional decline, or increased healthcare contact. The stronger associations observed within the first year after diagnosis suggest a period of heightened systemic vulnerability, emphasizing the need for closer clinical monitoring during early disease phases.

Diabetes showed consistent associations both before and after BP diagnosis, supporting prior evidence of a bidirectional relationship.^18,19^ Chronic systemic inflammation may contribute to metabolic dysregulation, while corticosteroid therapy may exacerbate glucose intolerance. In addition, previous research has linked dipeptidyl peptidase-4 inhibitors (DPP-4i) to increased BP risk,^20,21^ suggesting that pharmacologic exposure may partly contribute to this association. Although medication data were not available in our registry analysis, these findings underscore the complex interplay between metabolic disease and BP pathogenesis.

Our analysis highlights a significant association between BP and neurological disorders, reinforcing previous findings that suggest a link between neurodegenerative conditions and BP. Dementia emerged as one of the most prominent neurological associations, with increased prevalence prior to BP diagnosis and sustained incidence afterward. These findings align with prior research demonstrating a strong association between BP and neurodegenerative disorders. A recent systematic review and meta-analysis found a substantially elevated association of all-cause dementia in BP patients, with pooled odds ratios of 4.25 (95% CI 2.73–6.61) in case-control studies and HR of 1.41 (95% CI 1.20–1.66) from cohort studies.^22^ The association between BP and dementia has been documented before, with proposed mechanisms including neuro-inflammation, shared autoantibodies, and blood-brain barrier dysfunction.^23^ Additionally, Parkinson’s disease demonstrated a similar pattern, supporting the growing evidence of a bidirectional relationship between neurodegeneration and BP.^24^

Another comprehensive study highlighted that 26.4% to 55.8% of BP patients present with at least one neurological condition, such as stroke, dementia, or Parkinson’s disease, compared to 9.1% to 20.5% in control groups.^25^

The relationship between BP and epilepsy also remains a subject of interest. This is consistent with previous research showing a heightened prevalence of epilepsy in BP patients, particularly among those with preexisting neurodegenerative conditions.^23^ The role of neuro-inflammation in both conditions also supports the hypothesis that autoantibodies in BP may contribute to neuronal damage, reinforcing the need for neurological monitoring in BP patients.^26^

We observed that the HR for MS was elevated but with wide confidence intervals, and the number of observed cases was small, making this result inconclusive. These contrasts with previous large-scale studies that have consistently reported a strong association between MS and BP.^27^

Nonetheless, the elevated POR for MS suggests a strong association, with individuals diagnosed with BP being more than seven times as likely to have a history of MS compared to matched controls. Notably, this association remains substantial and statistically significant when examining diagnoses that occur more than one year before BP onset. The elevated POR values before BP onset suggest that MS may act as a predisposing factor or early autoimmune marker in BP pathogenesis. In a large population based case–control study from the UK, MS was strongly associated with the subsequent development of BP, with an adjusted odds ratio of 10.7 (95% CI: 2.8–40.2).^28^ The hypothesized mechanism involves chronic neuroinflammation and the presence of cross-reactive autoantibodies targeting both myelin and skin basement membrane components.^27^

We found an association between ALS and BP, particularly with ALS appearing before the onset of BP. Our findings align with earlier reports, such as those by Chosidow et al. (2000), who identified BP in 3 out of 168 ALS patients, and Nakane et al. (2016), who described six additional cases where BP developed, on average, 5.6 years after ALS diagnosis.^29,30^ Other case reports have proposed that damage to the nervous system in ALS might trigger an autoimmune response due to cross-reactivity with BP antigen 1.^29^ While these observations suggest a possible link between the two conditions, larger epidemiological studies are still necessary to confirm this association.

Psychiatric comorbidities, including unipolar/bipolar disease and suicide risk, were also elevated in BP patients. Research done by Egeberg et al. (2020) demonstrated a significantly increased prevalence of major depressive disorder and suicide attempts in BP patients.^31^ A recent systematic review and meta-analysis demonstrated that individuals with BP have a significantly higher risk of developing psychiatric disorders, including schizophrenia, with an odds ratio of 2.63 (95% CI: 2.03–3.39), further supporting the association between BP and severe psychiatric comorbidities.^32^ The reasons for this association may include chronic pain, pruritus, reduced quality of life, and potential psychiatric comorbidities.

The increased prevalence of stroke prior to BP diagnosis suggests that cerebrovascular events may act as potential triggers rather than merely consequences of systemic inflammation. A study from Taiwan reported that BP patients had a significantly increased risk of ischemic stroke compared to matched controls, with an adjusted hazard ratio of 2.37 (95% CI: 1.78–3.15), suggesting a potential role of endothelial dysfunction and systemic inflammation in BP pathogenesis.^33^ A study of stroke patients and matched controls revealed that anti-BP180 autoantibodies were detectable in 14% of stroke patients, compared to 5% of healthy controls, suggesting that post-stroke immune dysregulation may lead to basement membrane autoantibody formation and potentially trigger bullous pemphigoid.^34^ This aligns with our finding of a markedly increased stroke risk in the year preceding BP diagnosis, reinforcing the hypothesis that stroke may act as a predisposing factor for BP rather than solely being a consequence of chronic inflammation in BP patients. This finding is consistent with research indicating that chronic inflammatory skin diseases, including BP, are associated with an increased risk of thromboembolic events, likely due to persistent systemic inflammation and vascular endothelial dysfunction.^35^

Psoriasis, lichen planus, vitiligo, and alopecia areata were more prevalent in BP patients, suggesting potential shared autoimmune mechanisms and inflammatory pathways. These findings align with previous research that has linked BP to other skin diseases. An extensive population-based study found that psoriasis was significantly associated with an increased risk of BP, with an adjusted hazard ratio of 3.05 (95% CI: 2.10–4.43).^36^ Furthermore, our findings align with previous studies, including a recent case series, which reported an increased occurrence of BP-like disease in patients with lichen planus, particularly in the form of lichen planus pemphigoides. This overlap is thought to result from shared autoimmune mechanisms involving T-cell activation and basal keratinocyte injury.^37^

Furthermore, a study revealed that patients with vitiligo have an increased risk of developing BP, possibly due to autoantibody cross-reactivity against melanocyte and keratinocyte antigens.^38^ In our study, we observed a significant association between alopecia areata and BP prior to BP diagnosis. However, the current literature on this relationship is limited, with only a few isolated case reports suggesting possible overlap, and no large-scale epidemiological studies have confirmed this association to date. Among systemic autoimmune diseases, systemic sclerosis showed an influential association with BP. A relationship that has previously only been described in isolated case reports.^39^ While these individual cases suggest a possible clinical overlap, our findings provide broader support for a potential link between the two conditions, which may share underlying autoimmune mechanisms. Although rare, bullous lesions can occur in SLE through bullous SLE, and up to seven cases have been reported where SLE and classic BP coexisted.^40^ We observed an association between BP and the subsequent development of Sjögren’s Syndrome, particularly emerging more than one year after BP diagnosis. Current evidence on this relationship is limited to isolated case reports.^41^ To date, no large-scale epidemiological studies have established a consistent population-level association.

The present findings have direct implications for clinical practice. Dermatologists should recognize BP as a condition frequently embedded within a broader multimorbid profile, particularly in elderly patients. Baseline assessment at diagnosis should therefore extend beyond cutaneous evaluation and include structured review of neurological history, metabolic status, and psychiatric well-being.

### Strengths and limitations

This study has several limitations. Comorbidities were identified using registry-based diagnostic codes without access to detailed clinical records, which may result in misclassification. Information on disease severity, medication exposure, seasonality, and lifestyle factors was not available and could not be adjusted for, leaving the possibility of residual confounding. The retrospective design relies on the accuracy of registry data, and heterogeneity in disease severity may have influenced the results. Despite the high positive predictive value of BP diagnoses in the NPR, coding errors may occur.^16^ Additionally, the findings may not be directly generalizable to healthcare systems with different population structures or access to care.

Despite these limitations, the study’s strengths include its large, population-based design and use of validated national data. It is the most extensive study to explore comorbidities both before and after BP diagnosis, allowing for assessment of prodromal factors and long-term disease burden. The extended follow-up and focus on incident cases further strengthen the reliability of the findings.

Furthermore the Swedish healthcare setting provides a unique epidemiological context for studying BP-associated disease burden. Sweden has one of the highest reported incidences of BP in Europe, an aging population, and universal access to specialist care. The near-complete national coverage and high diagnostic validity of the NPR allow for comprehensive assessment of multimorbidity patterns at the population level. These features strengthen the generalizability of our findings within similar healthcare systems and underscore their relevance for healthcare planning and multidisciplinary management.

## Conclusion

This nationwide study demonstrates that BP is associated with a broad spectrum of systemic comorbidities occurring both before and after diagnosis. These findings support the view that BP represents a systemic disease embedded within age-related multimorbidity rather than a purely cutaneous disorder. Recognition of BP as part of a complex clinical profile should encourage proactive screening, interdisciplinary collaboration, and individualized follow-up strategies. Future research should clarify causal mechanisms and explore whether targeted preventive strategies can reduce morbidity and mortality in this vulnerable population.

## Data Availability

The data underlying this article will be shared on reasonable request to the corresponding author.

## References

[1] Genovese G Di Zenzo G Cozzani E , et al. New Insights Into the Pathogenesis of Bullous Pemphigoid: 2019 Update. Front Immunol 2019; 10: 1506. 10.3389/fimmu.2019.01506 31312206 PMC6614376

[2] Hertl M . Humoral and cellular autoimmunity in autoimmune bullous skin disorders. International Archives of Allergy and Immunology 2000; 122(2): 91–100. 10.1159/000024364 10878487

[3] Di Zenzo G Marazza G Borradori L . Bullous pemphigoid: Physiopathology, clinical features and management. Advances in Dermatology 2007; 23: 257–288. 10.1016/j.yadr.2007.07.013 18159905

[4] Brick KE Weaver CH Lohse CM , et al. Incidence of bullous pemphigoid and mortality of patients with bullous pemphigoid in Olmsted County, Minnesota, 1960 through 2009. Journal of the American Academy of Dermatology 2014; 71(1): 92–99. 10.1016/j.jaad.2014.02.030 24704091 PMC4324601

[5] Försti AK Jokelainen J Timonen M , et al. Increasing incidence of bullous pemphigoid in Northern Finland: a retrospective database study in Oulu University Hospital. British Journal of Dermatology 2014; 171(5): 1223–1226. 10.1111/bjd.13189 24934834

[6] Persson MSM Harman KE Vinogradova Y , et al. Incidence, prevalence, and mortality of bullous pemphigoid in England 1998–2017: A population-based cohort study. British Journal of Dermatology 2020; 184(1): 68–77. 10.1111/bjd.19022 32147814

[7] Thorslund K Seifert O Nilzén K , et al. Incidence of bullous pemphigoid in Sweden 2005–2012: A nationwide cohort study of 3,761 patients. Archives of Dermatological Research 2017; 309(9): 721–727. 10.1007/s00403-017-1778-4 28875235 PMC5648739

[8] Colbert RL Allen DM Eastwood D , et al. Mortality rate of bullous pemphigoid in a U.S. medical center. Journal of Investigative Dermatology 2004; 122(5): 1091–1095. 10.1111/j.0022-202X.2004.22504.x 15140208

[9] Roujeau JC Lok C Bastuji-Garin S , et al. High risk of death in elderly patients with extensive bullous pemphigoid. Archives of Dermatology 1998; 134(4): 465–469. 10.1001/archderm.134.4.465 9554299

[10] Korman NJ . Bullous pemphigoid: The latest in diagnosis, prognosis, and therapy. Archives of Dermatology 1998; 134(9): 1137–1141. 10.1001/archderm.134.9.1137 9762028

[11] Lee S Rastogi S Hsu DY , et al. Association of bullous pemphigoid and comorbid health conditions: A case–control study. Archives of Dermatological Research 2021; 313(5): 327–332. 10.1007/s00403-020-02100-2 32647978

[12] Ren Z Hsu DY Brieva J , et al. Hospitalization, inpatient burden and comorbidities associated with bullous pemphigoid in the U.S.A. British Journal of Dermatology 2017; 176(1): 87–99. 10.1111/bjd.14821 27343837

[13] Atzmony L Mimouni I Reiter O , et al. Association of bullous pemphigoid with malignancy: A systematic review and meta-analysis. Journal of the American Academy of Dermatology 2017; 77(4): 691–699. 10.1016/j.jaad.2017.05.006 28645646

[14] Albadri Z Thorslund K Häbel H , et al. Increased risk of squamous cell carcinoma of the skin and lymphoma among 5,739 patients with bullous pemphigoid: A Swedish nationwide cohort study. Acta Dermato-Venereologica 2020; 100(17): adv00289. 10.2340/00015555-3622 32852559 PMC9274914

[15] Ludvigsson JF Andersson E Ekbom A , et al. External review and validation of the Swedish national inpatient register. BMC Public Health 2011; 11: 450. 10.1186/1471-2458-11-450 21658213 PMC3142234

[16] Grönhagen C Nilzén K Seifert O , et al. Bullous pemphigoid: Validation of the National Patient Register in two counties in Sweden, 2001 to 2012. Acta Dermato-Venereologica 2017; 97(1): 32–35. 10.2340/00015555-2456 27171523

[17] Langan SM Smeeth L Hubbard R , et al. Bullous pemphigoid and pemphigus vulgaris: incidence and mortality in the UK: Population-based cohort study. BMJ 2017; 337: a180. 10.1136/bmj.a180 PMC2483869 18614511

[18] Jiirasutat N Pongchareon P Weschawalit S . Bullous pemphigoid and diabetes mellitus: a systematic review and meta-analysis. Int J Dermatol 2024; 63: 572–579. 10.1111/ijd.16970 38217028

[19] Chuang TY Korkij W Soltani K , et al. Increased frequency of diabetes mellitus in patients with bullous pemphigoid: A case–control study. Journal of the American Academy of Dermatology 1984; 11(6): 1099–1102. 10.1016/s0190-9622(84)70266-0 6392367

[20] Tasanen K Varpuluoma O Nishie W . Dipeptidyl Peptidase-4 Inhibitor Associated Bullous Pemphigoid. Frontiers in Immunology 2019; 10: 1238. 10.3389/fimmu.2019.01238 31275298 PMC6593303

[21] Kridin K Bergman R . Association of Bullous Pemphigoid With Dipeptidyl-Peptidase 4 Inhibitors in Patients With Diabetes: Estimating the Risk of the New Agents and Characterizing the Patients. JAMA dermatology 2018; 154(10): 1152–1158. 10.1001/jamadermatol.2018.2352 30090931 PMC6233738

[22] Zhou Q Xiong Z Yang D , et al. The association between bullous pemphigoid and cognitive outcomes in middle-aged and older adults: A systematic review and meta-analysis. PLoS ONE 2023; 18(11): e0295135. 10.1371/journal.pone.0295135 38033098 PMC10688758

[23] Chen J Li L Chen J , et al. Sera of elderly bullous pemphigoid patients with associated neurological diseases recognize bullous pemphigoid antigens in the human brain. Gerontology 2011; 57(3): 211–216. 10.1159/000315393 20664178

[24] Lai YC Yew YW Lambert WC . Bullous pemphigoid and its association with neurological diseases: a systematic review and meta-analysis. J Eur Acad Dermatol Venereol 2016; 30(12): 2007–2015. 10.1111/jdv.13660 27599898

[25] Huttelmaier J Benoit S Goebeler M . Comorbidity in bullous pemphigoid: Update and clinical implications. Frontiers in Immunology 2023; 14: 1196999. 10.3389/fimmu.2023.1196999 37457698 PMC10346857

[26] Pietkiewicz P Gornowicz-Porowska J Bowszyc-Dmochowska M , et al. Bullous pemphigoid and neurodegenerative diseases: A study in a setting of a Central European university dermatology department. Clinical, Cosmetic and Investigational Dermatology 2016; 9: 659–663. 10.1007/s40520-015-0459-4 PMC4930474 26420424

[27] Kibsgaard L Rasmussen M Lamberg A , et al. Increased frequency of multiple sclerosis among patients with bullous pemphigoid: A population-based cohort study anchored around the diagnosis of bullous pemphigoid. British Journal of Dermatology 2017; 176(6): 1486–1491. 10.1111/bjd.15405 28235244

[28] Langan SM Groves RW West J . The relationship between neurological disease and bullous pemphigoid: A population-based case-control study. Journal of Investigative Dermatology 2011; 131(3): 631–636. 10.1038/jid.2010.357 21085189

[29] Chosidow O Doppler V Bensimon G , et al. Bullous pemphigoid and amyotrophic lateral sclerosis: a new clue for understanding the bullous disease? Arch Dermatol 2000; 136(4): 521–524. 10.1001/archderm.136.4.521 10768651

[30] Nakane S Izumi Y Oda M , et al. A Potential Link between Amyotrophic Lateral Sclerosis and Bullous Pemphigoid: Six New Cases and a Systematic Review of the Literature. Intern Med 2016; 55(15): 1985–1990. 10.2169/internalmedicine.55.5578 27477403

[31] Rania M Petersen LV Benros ME , et al. Psychiatric comorbidity in individuals with bullous pemphigoid and all bullous disorders in the Danish national registers. BMC Psychiatry 2020; 20(1): 411. 10.1186/s12888-020-02810-x 32819315 PMC7439544

[32] Huang IH Wu PC Liu CW , et al. Association between bullous pemphigoid and psychiatric disorders: A systematic review and meta-analysis. J Dtsch Dermatol Ges 2022; 20(10): 1305–1312. 10.1111/ddg.14852 36108333

[33] Yang Y-W Chen Y-H Xirasagar S , et al. Increased risk of stroke in patients with bullous pemphigoid: A population-based follow-up study. Stroke 2011; 42(2): 319–323. 10.1161/STROKEAHA.110.596361 21164122

[34] Wang J Liu H Wang Z , et al. Analysis of the autoimmune response to BP180 in Chinese stroke patients. An Bras Dermatol 2023; 98(1): 13–16. 10.1016/j.abd.2022.01.012 36456305 PMC9837628

[35] Schneeweiss MC Merola JF Wyss R , et al. Venous Thromboembolism in Patients With Bullous Pemphigoid. JAMA Dermatol 2023; 159(7): 750–756. 10.1001/jamadermatol.2023.1461 37285147 PMC10248807

[36] Ho YH Hu HY Chang YT , et al. Psoriasis is associated with increased risk of bullous pemphigoid: A nationwide population-based cohort study in Taiwan. J Dermatol 2019; 46(7): 604–609. 10.1111/1346-8138.14902 31062428

[37] Combemale P Bouaziz JD Chosidow O , et al. Lichen planus pemphigoides with predominant mucous membrane involvement: a series of 12 patients and a literature review. Front Immunol 2024; 15: 1243566. doi:10.3389/fimmu.2024.1243566. 38686381 PMC11057232

[38] Oka M Fukumoto T . Bullous Pemphigoid in Which Eruption Developed Exclusively on Preexisting Eruption of Vitiligo Vulgaris. Case Rep Dermatol 2020; 12(1): 33–36. 10.1159/000505829 32110207 PMC7036583

[39] Sherber NS Wigley FM Anhalt GJ . Bullous pemphigoid in a patient with systemic sclerosis (scleroderma). J Rheumatol 2006; 33(10): 2098. 17014027

[40] Lance NJ Blaszak W Swartz TJ . Bullous skin lesions in systemic lupus erythematosus. Seminars in Arthritis and Rheumatism 1991; 20(6): 396–404. 10.1016/0049-0172(91)90015-r

[41] García Briz MI Prats Máñez A García Ruiz R , et al. Livedoid vasculopathy in a patient with bullous pemphigoid and primary Sjögren’s syndrome. Reumatol Clin (Engl Ed) 2020; 16(2 Pt 2): 189–190. 10.1016/j.reuma.2018.01.013 29526676

